# Microencapsulated Blend of Thyme, Oregano, and Ajwain Essential Oils Enhances Broiler Productivity by Modulating Gut Morphology and Nutrient Digestibility

**DOI:** 10.3390/vetsci13090898

**Published:** 2026-09-01

**Authors:** Maryam Sahraei, Reza Mahdavi, Soudabeh Moradi, Leili Jamshidi

**Affiliations:** Department of Animal Science, College of Agriculture and Natural Resources, Razi University, Kermanshah 6715685438, Irans.moradi@razi.ac.ir (S.M.);

**Keywords:** thyme, oregano, ajwain, immune response, antioxidant status

## Abstract

The global poultry industry seeks sustainable alternatives to antibiotic growth promoters due to concerns regarding antimicrobial resistance and chemical residues. This trial examined the physiological and productive impacts of a microencapsulated blend of thyme, oregano, and ajwain essential oils (TOA-EOs) in broiler chickens. A total of 360 male Ross 308 broilers were assigned to four dietary treatments: a basal diet (NC), a basal diet with 50 mg/kg bacitracin (PC), and diets supplemented with either 0.75 or 1.5 g/kg of TOA-EOs. Results demonstrated that TOA-EO supplementation significantly reduced feed intake (*p* < 0.05) and improved feed conversion ratio (*p* < 0.05), particularly in the 0.75 g/kg group compared to NC. However, pairwise comparisons for body weight gain showed no significant differences among treatments based on Tukey’s HSD test, despite a significant overall ANOVA (*p* < 0.05). These performance benefits were linked to enhanced jejunal morphology, characterized by significantly increased villus height, surface area, and villus height to crypt depth ratio (*p* < 0.05). Furthermore, the apparent ileal digestibility of crude protein was significantly enhanced (*p* < 0.05), although serum antioxidant status and immune response against Newcastle disease virus were not significantly influenced (*p* > 0.05). In conclusion, microencapsulated TOA-EOs effectively promote broiler growth performance by refining intestinal structure and enhancing nutrient digestibility, serving as a potent and sustainable alternative to traditional antibiotics.

## Simple Summary

The poultry industry is limiting antibiotic use due to concerns over drug resistance and chemical residues in meat. This study evaluated a natural alternative: a protected mixture of thyme, oregano and ajwain plant oils. These oils were encased in a special coating to ensure they reached the broiler’s digestive system safely. Our findings showed that chickens receiving this herbal supplement used their feed more efficiently. These improvements were driven by a healthier gut structure, which significantly increased the broiler chicks’ ability to digest and absorb protein. While the supplement did not change the chicks’ immune system or antioxidant status, it proved to be a highly effective growth promoter. This research provides a sustainable way for farmers to produce healthy meat without antibiotics. By ensuring food safety while maintaining high productivity, this natural solution benefits both the poultry industry and consumer health.

## 1. Introduction

The global poultry industry has moved toward restricting antibiotic growth promoters due to escalating concerns regarding antimicrobial resistance and the presence of chemical residues in meat [1]. Since the European Union’s ban on prophylactic antibiotic use, researchers have explored sustainable alternatives, including probiotics, prebiotics and phytogenic feed additives. These natural solutions aim to maintain high productivity while ensuring food safety for consumers [2].

One of effective phytogenics, oregano (*Origanum vulgare*) is a versatile herb characterized by carvacrol, thymol and rosmarinic acid. Oregano essential oil is instrumental in performance improvement, specifically by enhancing feed conversion [3,4,5]. Moreover, its potent antioxidant and immunomodulatory properties may prevent lipid oxidation [2,4]. Similarly, thyme (*Thymus vulgaris*) is highly valued for its rich phenolic content, primarily thymol and carvacrol, alongside p-cymene and γ-terpinene [6,7]. Thyme essential oils have been shown to enhance growth performance by increasing the secretion of endogenous digestive enzymes [1,8]. Additionally, its potent antioxidant and antimicrobial activities help stabilize the gut environment, reducing the colonization of pathogenic bacteria [2,9]. Furthermore, ajwain (*Trachyspermum ammi*) is a prominent medicinal plant containing high concentrations of thymol (35–60%), γ-terpinene and p-cymene [10,11]. Research indicates that ajwain supplementation can significantly improve BWG and immune competence in broiler chickens [10,12]. These benefits are often associated with a more balanced ileal microbial population and reduced physiological stress markers [13,14].

Despite these benefits, the practical application of essential oils is often limited by their high volatility and rapid degradation in the upper gastrointestinal tract [15,16]. The innovation of this research lies in the application of a synergistic blend of these three herbs encapsulated within a chitosan-based matrix. The complete formulation was microencapsulated with chitosan as a natural polymeric coating through a specialized spraying and cooling procedure. This encapsulation approach was intended to improve the stability of volatile bioactive constituents, particularly thymol and carvacrol, against the acidic conditions encountered in the upper gastrointestinal tract, and to enable targeted release of active compounds in the intestine of poultry [17,18]. This delivery system is crucial for maintaining mucosal structure and ensuring bioactive molecules reach their site of action effectively [18,19]. Because combinations of different herbs may yield enhanced benefits through combined or additive effects, there is a critical need to investigate this specific novel formulation. Consequently, the present study was conducted to investigate the potential synergistic effects of an encapsulated thyme, oregano and ajwain blend on growth performance, nutrient digestibility, intestinal morphology, antioxidant status and the immune system in broilers.

The complete formulation was microencapsulated with chitosan as a natural polymeric coating. This encapsulation approach was intended to improve the stability of volatile bioactive constituents, particularly thymol and carvacrol, to the acidic conditions encountered in the upper gastrointestinal tract.

## 2. Materials and Methods

### 2.1. Experimental Design, Animals and Diet

All procedures involving animals were conducted in accordance with the ethical standards approved by the Animal Ethics Committee of the Department of Animal Science, Razi University, Iran. In total, 360 male Ross 308 broiler chicks, one day of age, were allocated randomly to four dietary treatments. Each treatment consisted of six replicate floor pens, with 15 broilers housed in each pen. The dietary treatments included a negative control (NC), a positive control (PC) containing bacitracin citrate (10%) at 0.5 g/kg of diet and two additional treatments in which the NC diet was supplemented with 0.75 or 1.5 g/kg of a microencapsulated blend of thyme, oregano and ajwain. The commercial product used as the phytogenic additive (Noviherb^®^; Holding PARSA, Qom, Iran) consisted of a combined formulation designed to provide complementary phytogenic activities. The phytogenic fraction comprised essential oils derived from thyme (*Thymus vulgaris* L.), oregano (*Origanum vulgare* L.) and ajwain (*Trachyspermum ammi*), with each component incorporated at 2%. The microencapsulation process involved a specialized spraying and cooling procedure that generated a protective coating around the active components and was designed to promote their gradual release in the small intestine. Such protection may limit premature oxidation and degradation of the encapsulated compounds and thereby improve their delivery to the intestinal tract.

The feeding trial was conducted for 42 days in an environmentally controlled poultry house using floor pens measuring 1.4 × 1.5 m and containing deep litter, with a stocking density of approximately 7 chicks per square meter (15 broilers per pen). During the first 48 h post-hatch, continuous lighting (24 h) was provided to stimulate feed and water intake. Thereafter, the dark period was gradually increased by 0.5 h per day over a period of 6 days until reaching 3 h of darkness and 21 h of light per day. This lighting schedule was then maintained constant until the end of the experiment. The house temperature was gradually reduced according to standard recommendations; it was maintained at 33–35 °C during the first week and then decreased by 2–3 °C each subsequent week until reaching 22–24 °C at the end of the experiment on day 42. Relative humidity was kept between 55% and 65% throughout the study. Temperature and humidity were monitored daily using digital thermohygrometers placed at broiler height and distributed evenly within the house. Tunnel ventilation was provided to ensure adequate air exchange and to prevent ammonia accumulation, with ventilation rates adjusted based on ambient temperature and chick age. Throughout the experimental period, broilers received mash diets formulated to meet the recommended nutrient requirements of Ross 308 broilers and had unrestricted access to both feed and drinking water. The ingredient proportions and analyzed nutrient composition of the experimental diets are presented in Table 1.

### 2.2. Production Performance

Growth performance was monitored during the entire 42-day feeding trial. For each replicate, body weight gain (BWG) was determined from the difference between the collective body weight recorded at the beginning and end of each assessment period. Feed intake (FI) was estimated by subtracting the amount of feed remaining at the end of the respective period from the total quantity of feed provided to each replicate. To account for mortality, FI was adjusted according to the body weight of chickens that died during each experimental period. Feed conversion ratio (FCR) was subsequently calculated for each replicate by dividing mortality-adjusted FI by the corresponding BWG.

### 2.3. Intestinal Morphology

At the completion of the 42-day trial, a single broiler from each replicate was chosen for assessment of intestinal morphology, resulting in six chicks evaluated per dietary treatment. Selection was based on the individual broiler whose body weight most closely approximated the mean body weight of its respective replicate. Broilers were euthanized by jugular venesection, after which a 2-cm portion of the jejunum was excised approximately 5 cm proximal to Meckel’s diverticulum. The collected tissue was immediately immersed in 10% neutral-buffered formalin for fixation. For histological processing, the jejunal specimens were dehydrated using progressively increasing concentrations of ethanol, cleared with xylene and subsequently embedded in paraffin. Paraffin blocks were sectioned at a thickness of 5 μm using a rotary microtome (CUT4055, microTec Laborgeräte GmbH, Walldorf, Germany). The sections were stained with hematoxylin and eosin (H&E) and examined microscopically. Digital micrographs were obtained using an Olympus BX51TF light microscope (Olympus, Tokyo, Japan) coupled with a computer-assisted image analysis system. Villus height (VH), villus width (VW) and crypt depth (CD) were measured from the digital images. Villus surface area (VSA) was estimated following the approach described by Sakamoto et al. [20], according to the equation: VSA = 2π × (VW/2) × VH, where π = 3.14.

### 2.4. Antioxidant Parameters and Antibody Titers Against Newcastle Disease Virus

The humoral immune response to Newcastle disease virus (NDV) vaccination was assessed by monitoring serum antibody titers. On day 30 of the experiment, all chicks were vaccinated against NDV through the drinking water using a live CEVAC^®^ NEW L vaccine containing the La Sota strain (CEVA-Phylaxia, Budapest, Hungary). Blood samples (2 mL) were obtained from one designated broiler in each replicate on days 28 and 40. The initial samples were collected before vaccination to establish baseline NDV antibody levels and verify the similarity of humoral immune status among treatments. The second sampling was conducted 10 days after vaccination to assess the antibody response elicited by the vaccine. Following collection, blood was centrifuged at 3000 rpm for 15 min and the recovered serum was stored at −20 °C until analysis. NDV-specific antibody titers were quantified using the standard hemagglutination inhibition (HI) test.

Antioxidant status was evaluated at the conclusion of the 42-day experiment using one chick from each replicate, selected according to the criterion of body weight being closest to the corresponding replicate mean. Two blood samples, each 4 mL, were collected from each selected broiler. One aliquot was placed in an EDTA-containing tube for whole-blood analyses, whereas the second was collected into a tube without anticoagulant to obtain serum. Serum samples were separated by centrifugation and maintained at −20 °C until biochemical analyses. Serum malondialdehyde (MDA) concentration was quantified using the procedure of Ohkawa et al. [21], which involves thiobarbituric acid reaction, extraction with butanol and subsequent spectrophotometric determination. Superoxide dismutase (SOD) activity was analyzed using the RANSOD commercial assay kit, while glutathione peroxidase (GPx) activity was measured in whole blood using the RANSEL kit (Randox Laboratories, Crumlin, UK). Serum total antioxidant capacity (TAC) was determined following the methodology reported by Mahmoud and Hijazi [22]. All biochemical parameters were quantified spectrophotometrically in accordance with the instructions provided for the respective analytical procedures.

### 2.5. Nutrient Digestibility

Apparent ileal digestibility (AID) of dry matter (DM), crude protein (CP), ash, calcium (Ca) and phosphorus (P) was estimated using titanium dioxide (TiO_2_) as an indigestible dietary marker. The marker was incorporated into the experimental diets at a concentration of 0.5% (5 g/kg) for the final 72 h of the feeding trial. Titanium dioxide (Merck, Darmstadt, Germany) was thoroughly mixed with the diets before they were offered to the broilers. At the conclusion of the experiment, two chicks from each replicate were euthanized and ileal digesta were collected from the portion of the ileum located between Meckel’s diverticulum and a point 5 cm proximal to the ileocecal junction. Samples obtained from the two broilers within each replicate were combined to form one representative sample and stored at −20 °C pending laboratory analysis. In addition, approximately 1 kg of each experimental diet fed during the marker period was retained for subsequent chemical analyses. Prior to chemical analysis, the ileal digesta samples were freeze-dried and finely ground to ensure sample homogeneity. The DM content of both feed and digesta was determined by drying at 105 °C to a constant weight according to AOAC [23]. CP was quantified using the Kjeldahl procedure, with nitrogen values converted to CP using a factor of 6.25 [23]. Ash concentration was determined by combustion in a muffle furnace at 600 °C for 2 h. Ca concentrations were analyzed by atomic absorption spectrophotometry using a ZEEnit 700p instrument (Analytik Jena, Jena-Göschwitz, Germany), whereas total P was quantified colorimetrically by the ammonium vanado-molybdate procedure using a Unico S2100 spectrophotometer (UNICO, Fairfield, NJ, USA). Titanium dioxide concentrations in the diets and ileal digesta were measured by the colorimetric procedure described by Short et al. [24]. The AID of each nutrient was calculated using the marker ratio method according to the following equation:

$${AID}_{nutrient}=1-\left\{\left(\frac{{Ti}_{Diet}}{{Ti}_{Digesta}}\right)\times \left(\frac{{Nutrient}_{Digesta}}{{Nutrient}_{Diet}}\right)\right\}$$

where [Ti]_Diet and [Ti]_Digestadenote the titanium dioxide concentrations in the diet and ileal digesta, respectively, while [Nutrient]_Dietand [Nutrient]_Digesta represent the concentrations of the respective nutrient in the diet and ileal digesta, respectively.

### 2.6. Statistical Analysis

Statistical analyses were conducted using the GLM procedure of SAS software, version 9.4 (SAS Institute Inc., Cary, NC, USA). Dietary treatment was specified as the fixed effect in the statistical model. For growth performance traits, including BWG, FI and FCR, the pen was defined as the experimental unit. In contrast, for jejunal histomorphometric measurements, antioxidant-related variables, NDV-specific antibody titers and apparent ileal digestibility coefficients, the sampled chick within each replicate was considered the experimental unit. Treatment means were compared using Tukey’s Honestly Significant Difference (HSD) test when the overall treatment effect reached statistical significance (*p* < 0.05). Trends toward significance were defined as 0.05 < *p* < 0.10 and were noted where applicable. In addition, orthogonal polynomial contrasts were used to determine whether increasing dietary concentrations of the microencapsulated phytogenic blend resulted in significant linear or quadratic responses. For orthogonal polynomial contrast analysis, the PC group was excluded, and the analysis was performed only on the NC and the two TOA-EO supplementation levels to evaluate linear and quadratic dose–response trends. Data are expressed as treatment means accompanied by the pooled standard error of the mean (SEM).

## 3. Results

### 3.1. Production Performance

The effects of dietary treatments on the growth performance parameters of broiler chickens are summarized in Table 2. The results indicated that BWG was significantly influenced by the experimental diets (*p* < 0.05). Broilers supplemented with 1.5 g/kg of TOA-EOs achieved the highest weight gain (2935 g) compared to the PC group (2821 g); however, Tukey’s post hoc test revealed no significant pairwise differences among treatments, and no significant linear or quadratic trends were observed for this parameter (*p* > 0.05). In contrast, FI was significantly affected by the dietary inclusion of microencapsulated essential oils (*p* < 0.05). Broiler chicks in the 0.75 g/kg TOA-EO group consumed significantly less feed than the NC group (4512 g vs. 4837 g; *p* < 0.05, based on Tukey’s HSD test) and trend analysis showed a significant quadratic reduction in FI (P-quadratic < 0.05). Based on the regression analysis, the optimum dietary inclusion level of TOA-EOs for minimizing FI was determined to be 0.837 g/kg. Furthermore, the FCR was significantly improved by TOA-EO supplementation (*p* < 0.05). The lowest FCR (1.59) was observed in the 0.75 g/kg group compared to the NC group (1.70; *p* < 0.05, based on Tukey’s HSD test). Trend analysis for FCR demonstrated both significant linear and quadratic (*p* < 0.05) responses. Regression modeling revealed that the optimum supplementation dose for optimizing the FCR was 1.07 g/kg.

### 3.2. Antioxidant Parameters and Antibody Titers Against NDV

The effects of dietary supplementation with a microencapsulated mixture of thyme, oregano and ajwain essential oils on antioxidant biomarkers and humoral immune response are presented in Table 3. Statistical analysis revealed that the antioxidant status of broiler chickens was not significantly influenced by the experimental diets (*p* > 0.05). Specifically, the levels of TAC, MDA and the activities of SOD and GPX remained comparable across all treatment groups. Furthermore, no significant linear or quadratic trends were observed for any of the evaluated antioxidant parameters as the inclusion level of microencapsulated TOA-EOs increased (*p* > 0.05). Regarding the humoral immune response, the antibody titers against NDV measured at 28 and 40 days of age were not significantly affected by the dietary treatments (*p* > 0.05). The mean antibody titers at 40 days of age (10 days post-vaccination) showed numerical variations, ranging from 5.33 in the NC and 1.5 g/kg groups to 5.83 in the PC and 0.75 g/kg groups, without reaching statistical significance. However, an evaluation of the difference in titers between the two measurement periods revealed a notable tendency toward a quadratic response (*p* = 0.076), with the 0.75 g/kg inclusion level demonstrating the greatest numerical increase (1.50) compared to the negative control (0.667).

### 3.3. Intestinal Morphology

The data representing the effects of dietary inclusion of microencapsulated TOA-EOs on the jejunal histomorphology of broiler chickens are summarized in Table 4. The results indicated that VH was significantly influenced by the dietary treatments (*p* < 0.05). Broilers supplemented with 1.5 g/kg of microencapsulated TOA-EOs exhibited the highest VH (908 μm) compared to the NC (817 μm) and PC (804 μm) groups. Trend analysis for VH revealed a significant quadratic response (P-quadratic < 0.05) and a marginal linear tendency (P-linear = 0.052) as the supplementation levels increased. In terms of VW, although no significant differences were observed among the experimental groups via ANOVA (*p* > 0.05), a marginal linear tendency (P-linear < 0.05) was detected, suggesting a potential increase in width with higher doses of the additive. While the overall effect of treatments on CD was not statistically significant (*p* > 0.05), the analysis revealed a significant quadratic response (P-quadratic < 0.05), indicating a non-linear modulation of CD by the microencapsulated essential oils. Regression modeling revealed that the optimum supplementation dose for optimizing the CD was 0.876 g/kg. Furthermore, the VH/CD ratio was significantly improved by the TOA-EO supplementation (*p* < 0.05). The highest ratio (6.10) was achieved in the 1.5 g/kg group, while the lowest was observed in the NC group (5.25). Tukey’s test indicated that the VH/CD ratio in the 1.5 g/kg group was significantly higher than the NC group (*p* < 0.05). This parameter exhibited highly significant linear and quadratic (*p* < 0.05) responses to the increasing levels of the blend. Similarly, the VSA was significantly enhanced by the experimental diets (*p* < 0.05). Supplementation with 1.5 g/kg of microencapsulated TOA-EOs resulted in the largest surface area (0.476 mm^2^) which was significantly higher than the NC group (0.397 mm^2^; *p* < 0.05, based on Tukey’s HSD test). Trend analysis for VSA demonstrated significant linear and quadratic (*p* < 0.05) trends.

### 3.4. Nutrient Digestibility

The effects of dietary inclusion of a microencapsulated TOA-EOs on the apparent ileal nutrient digestibility of broiler chickens are summarized in Table 5. The results indicated that the apparent ileal digestibility of CP was significantly enhanced by the experimental diets (*p* < 0.05). Broilers supplemented with 1.5 g/kg of TOA-EOs achieved the highest CP digestibility (84.1%) compared to the NC (76.3%) and PC (74.4%) groups. Trend analysis for CP digestibility demonstrated both highly significant linear and quadratic (*p* < 0.05) responses to increasing levels of the microencapsulated blend. Based on regression analysis, the optimum dietary inclusion level of TOA-EOs for maximizing CP digestibility was determined to be 1.34 g/kg. Regarding ash digestibility, although the overall treatment effect showed a tendency toward significance (P-ANOVA = 0.067), trend analysis revealed a significant linear improvement (*p* ≤ 0.05) as the supplementation levels of TOA-EOs increased from 0 to 1.5 g/kg. Similarly, while the ANOVA for DM digestibility did not reach statistical significance (*p* > 0.05), a marginal linear tendency (*p* = 0.073) was detected, suggesting a potential enhancement in DM utilization with higher inclusion rates. In contrast, the apparent ileal digestibility of minerals, including Ca and P, was not significantly affected by the dietary inclusion of microencapsulated essential oils (*p* > 0.05). No significant linear or quadratic trends were observed for these minerals (*p* > 0.05).

## 4. Discussion

Dietary supplementation with TOA-EOs significantly enhanced BWG and optimized the FCR in broiler chickens during the 42-day experimental period. These findings align with previous research demonstrating the growth-promoting potential of thyme [1,7,9], oregano [3,4] and ajwain [10]. The synergistic efficacy of this polyherbal blend is consistent with studies indicating that combining these specific phytogenics yields superior productive outcomes compared to single-plant additives [25,26]. The improvement in growth performance in the current study was directly supported by significant enhancements in intestinal morphology quantified in this trial (Table 4). Specifically, the 1.5 g/kg TOA-EO inclusion level significantly increased VH, VH/CD and VSA in the jejunum compared to the control group. Essential oils rich in thymol and carvacrol have been shown to increase VH and the VH/CD, which expands the mucosal surface area available for nutrient absorption [2,3,14]. Furthermore, the higher VH/CD ratio observed in the supplemented groups serves as an indicator of a more stable intestinal milieu and an accelerated rate of cell maturation. This physiological shift likely reduces the energy and nutrient demands required for the maintenance of gut tissues, thereby allowing more resources to be redirected toward muscle development and overall body growth [26,27]. This improved morphology is closely linked to the significant enhancement in CP digestibility observed in the TOA-EO groups. The higher nutrient retention observed in the TOA-EO groups provided the essential building blocks for superior muscle deposition. The improved performance may be attributed to phenolic compounds stimulating pancreatic and intestinal enzymes (protease, amylase), enhancing nutrient breakdown and nitrogen retention [1,7,8]. The chitosan-based microencapsulation system likely provided superior protection for volatile compounds, enabling targeted release in the distal gut to maintain mucosal integrity [15,17,18]. Our results contrast with studies reporting no performance effects from these herbs [28,29]. These discrepancies may reflect differences in geographical origin, chemotypes, plant part used, and additive form, all affecting phenolic concentrations and bioactivity [2,30]. Additionally, broiler responses are inclusion level-dependent, and suboptimal dosages may fail to elicit benefits [26,30]. In our trial, microencapsulation was likely the decisive factor in overcoming volatility and ensuring consistent physiological benefits.

Dietary TOA-EOs did not significantly affect serum antioxidant markers, consistent with previous studies reporting limited systemic effects [3,31], though contrasting with reports of enhanced antioxidant activity [7,15,32,33,34,35,36,37]. These discrepancies may be attributed to several factors: the inclusion levels used in our trial may have been below the threshold for systemic response [3]; antioxidant parameters were measured in serum rather than intestinal mucosa, muscle, or liver where bioactive compounds may be selectively retained [15,34]; and the absence of oxidative or environmental stressors during the experimental period may have rendered exogenous supplementation unnecessary for maintaining redox homeostasis [35].

Although NDV antibody titers did not differ significantly among treatments at 28 and 40 days, the change in titers between these ages showed a quadratic trend toward significance, suggesting a potential priming effect of TOA-EOs on humoral immune competency. This numerical enhancement aligns with reports that phenolic monoterpenes such as thymol and carvacrol can stimulate lymphocyte proliferation, macrophage activity, and upregulation of immune-related genes including Avian beta-defensin 1 and Mucin 2 in the intestinal mucosa [1,3,5,25,35,36,37,38,39]. Additionally, thyme supplementation has been shown to improve cytokine expression (IFN-γ, IL-10), which supports T-helper cell activation and B-lymphocyte maturation into antibody-secreting plasma cells [15,25].

In the current study, jejunal morphometric indices, including VH, VH/CD and VSA, exhibited significant linear and quadratic increases in response to rising TOA-EO inclusion levels. An expanded surface area and taller villi likely provide a greater physical environment for the deployment of nutrient transporters and brush border enzymes. This structural optimization probably maximized the capacity of the small intestine to handle the final stages of nutrient digestion. Our morphological results are consistent with the findings of several researchers [4,5,10,14,37]. The beneficial changes in gut morphology are mediated through multiple mechanisms. Phenolic monoterpenes (thymol and carvacrol) exert antimicrobial effects against enteric pathogens, reducing enterocyte turnover and promoting longer villi [1,2,4,40]. These compounds also stimulate mucin secretion from goblet cells, protecting villi from inflammatory erosion [2]. At the molecular level, they inhibit NF-κB and MAPK signaling pathways, downregulating pro-inflammatory cytokines (TNF-α, IL-1β, IL-6) and preventing mucosal injury [32,39,41]. This suppression is linked to upregulation of tight junction proteins (occludin, claudin-1, ZO-1) and MUC2, stabilizing the intestinal barrier against pathogen-induced erosion and oxidative apoptosis [3,35,39]. By mitigating subclinical inflammation and preserving barrier integrity, these phytogenics support taller villi, reduced enterocyte turnover, and optimized nutrient absorption while minimizing resources diverted to tissue repair [26,27]. It should be acknowledged that the geometric equation used to estimate villus surface area [20] assumes a simplified cylindrical structure and was originally developed in rats. While this model provides a useful comparative estimate, it may not fully capture the diverse morphological variations of broiler intestinal villi.

Our findings indicate selective enhancement in CP and ash digestibility following TOA-EO supplementation, consistent with previous studies reporting improved organic matter and protein digestibility with thyme, herbal blends, and probiotic–phytogenic combinations [25,26,27,42]. This improvement is supported by our histological measurements, which showed significant expansion in jejunal VH and VSA in supplemented chicks. This expanded surface area likely increases brush-border enzyme secretion and provides greater physical space for nutrient transporter insertion, thereby maximizing intestinal absorptive capacity [7,8,26,27]. Several biological mechanisms may mediate these effects. First, phenolic monoterpenes stimulate secretion of endogenous digestive enzymes including protease and trypsin [1,7]. Molecular evidence suggests encapsulated essential oils can upregulate cholecystokinin gene transcription, triggering pancreatic protease synthesis and release [15]. Second, these compounds may modulate intestinal pH toward a more acidic state, optimizing proteolytic enzyme activity and enhancing protein hydrolysis [15,25]. Third, bioactive terpenes stimulate expression of MUC2 and tight junction proteins (occludin, zonula occludens-1), protecting villus structural integrity and expanding absorptive surface area [3,15,32,35,39]. By strengthening the mucosal barrier, TOA-EOs likely reduce energy and nutrient costs associated with intestinal inflammation and repair, redirecting amino acids toward muscle development and growth [26,27].

It should be noted that a control group receiving the microcapsule carrier alone was not included in this study. Therefore, we cannot completely exclude the possibility that the observed effects may have been influenced by the carrier material rather than solely by the active essential oil components.

## 5. Conclusions

Dietary supplementation of the microencapsulated TOA-EO blend improved broiler feed conversion ratio and body weight gain, with optimal efficacy at approximately 1.07 g/kg. These effects were associated with enhanced jejunal morphology (increased villus height, surface area, and villus height-to-crypt depth ratio) and improved apparent ileal crude protein digestibility, without adversely affecting systemic antioxidant or immune status. These findings suggest that the microencapsulated TOA-EO blend holds promise as a potential non-antibiotic growth-promoting strategy for broiler production; however, further research under commercial conditions and with appropriate carrier controls is warranted to confirm its practical applicability and long-term sustainability.

## Figures and Tables

**Table 1:** Ingredients and nutrient composition of the basal diets.

Ingredients (%)	Starter	Grower	Finisher
Corn	52.30	60.42	62.18
Soybean meal	39.80	32.70	30.50
Soybean oil	3.10	2.80	3.6
Dicalcium phosphate	2.30	1.80	1.7
Limestone	0.80	0.70	0.70
L-Lys HCL	0.27	0.30	0.21
DL-Met	0.38	0.35	0.30
L-Thr	0.16	0.16	0.10
Sodium bicarbonate	0.34	0.30	0.27
Salt	0.18	0.17	0.18
Mineral-Vitamin premix ^1^	0.27	0.22	0.20
Choline chloride	0.10	0.08	0.06
	100	100	100
**Calculated nutrient composition**			
ME (kcal/kg)	2950	3000	3070
CP (%)	22.00	19.50	18.50
Dig-Lys (%)	1.28	1.15	1.03
Dig-Met+Cys (%)	0.95	0.87	0.80
Dig-Thr (%)	0.86	0.77	0.69
Ca (%)	0.96	0.82	0.77
Available P (%)	0.48	0.40	0.37

^1^ Supplied per kilogram of diet: vitamin A, 9000 U; vitamin D3, 2000 U; vitamin E, 18 U; vitamin B12, 0.15 mg; riboflavin, 6.6 mg; calcium pantothenate, 10 mg; niacin, 30 mg; choline, 500 mg; biotin, 0.1 mg; thiamine, 1.8 mg; piridoxin, 3 mg; folic acid, 1 mg; vitamin K3, 2 mg; antioxidant (Ethoxyquin), 100 mg. zinc, 50 mg; manganese oxide, 100 mg; copper, 10 mg; Fe, 50 mg; I, 1 mg; Se, 0.2 mg.

**Table 2:** Effects of dietary inclusion of microencapsulated essential oils on growth performance of broiler chicks.

Items	NC	PC	TOA-EOs		SEM	*p*-Value		
			0.75 g/kg	1.5 g/kg		ANOVA	Linear	Quadratic
**BWG** (g; 1–42 days)	2855 ^a^	2821 ^a^	2835 ^a^	2935 ^a^	32.3	0.0002	0.492	0.548
**Feed intake** (g; 1–42 days)	4837 ^a^	4595 ^b^	4512 ^b^	4714 ^ab^	40.3	0.001	0.203	0.046
**FCR** (1–42 days)	1.70 ^a^	1.63 ^ab^	1.59 ^b^	1.61 ^ab^	0.017	0.011	0.048	0.010

A significant difference was considered at *p* < 0.05 based on Tukey’s HSD test. Trends toward significance (0.05 < *p* < 0.10) were also noted where applicable. Means within rows with different lower case letters differ significantly (*p* < 0.05) **NC**: basal diet without additives; **PC**: positive control (basal diet supplemented with 50 mg/kg bacitracin methylene disalicylate); **TOA-EOs**: basal diet supplemented with a microencapsulated mixture of Thyme, Oregano, and Ajwain. **BWG:** body weight gain, **FCR:** feed conversion ratio.

**Table 3:** Effects of dietary inclusion of microencapsulated essential oils on serum biomarkers and antibody against NDV of broiler chicks.

Items		NC	PC	TOA-EOs		SEM	*p*-Value		
				0.75 g/kg	1.5 g/kg		ANOVA	Linear	Quadratic
TAC (mmol/dL)		1.81	1.85	1.83	1.86	0.037	0.728	0.696	0.918
MDA (mmol/mL)		1.79	1.86	1.68	1.72	0.042	0.708	0.514	0.697
SOD (U/gHb)		2.40	2.57	2.33	2.31	0.042	0.255	0.423	0.732
GPx (U/gHb)		6.06	6.69	6.76	6.16	0.173	0.197	0.649	0.340
Antibody titer against NDV	28 days	4.67	4.50	4.33	4.50	0.120	0.918	0.479	0.562
	40 days	5.33	5.83	5.83	5.33	0.146	0.225	0.770	0.286
	Difference	0.667	1.33	1.50	0.83	0.133	0.106	0.425	0.076

A significant difference was considered at *p* < 0.05 based on Tukey’s HSD test. Trends toward significance (0.05 < *p* < 0.10) were also noted where applicable. **NC**: basal diet without additives; **PC**: positive control (basal diet supplemented with 50 mg/kg bacitracin methylene disalicylate); **TOA-EOs**: basal diet supplemented with a microencapsulated mixture of Thyme, Oregano, and Ajwain. **TAC:** Total antioxidant capacity, **MDA:** Malondialdehyde, **SOD:** superoxide dismutase, **GPx:** glutathione peroxidase, **NDV** titers: Newcastle disease virus antibody titers.

**Table 4:** Effects of dietary inclusion of microencapsulated essential oils on jejunal morphology of broiler chicks.

Items	NC	PC	TOA-EOs		SEM	*p*-Value		
			0.75 g/kg	1.5 g/kg		ANOVA	Linear	Quadratic
VH (μm)	817 ^b^	804 ^b^	824 ^b^	908 ^a^	13.7	0.002	0.052	0.045
VW (μm)	158	164	165	167	1.76	0.597	0.078	0.220
CD (μm)	158	147	145	151	1.70	0.132	0.113	0.042
VH/CD	5.25 ^b^	5.59 ^ab^	5.84 ^ab^	6.10 ^a^	0.094	0.030	0.001	0.005
VSA (mm^2^)	0.397 ^b^	0.412 ^b^	0.426 ^ab^	0.476 ^a^	0.009	0.045	0.003	0.006

A significant difference was considered at *p* < 0.05 based on Tukey’s HSD test. Trends toward significance (0.05 < *p* < 0.10) were also noted where applicable. Means within rows with different lower case letters differ significantly (*p* < 0.05). **NC**: basal diet without additives; **PC**: positive control (basal diet supplemented with 50 mg/kg bacitracin methylene disalicylate); **TOA-EOs**: basal diet supplemented with a microencapsulated mixture of Thyme, Oregano, and Ajwain. **VH**: villus height, **VW**: villus width, **CD**: crypt depth, **VH/CD**: villus height to crypt depth ratio, **VSA**: villus surface area.

**Table 5:** Effects of dietary inclusion of microencapsulated essential oils on apparent ileal nutrient digestibility of broiler chicks.

Items	NC	PC	TOA-EOs		SEM	*p*-Value		
			0.75 g/kg	1.5 g/kg		ANOVA	Linear	Quadratic
**DM**	75.8	75.8	79.3	78.6	0.591	0.133	0.073	0.114
**CP**	76.3 ^b^	74.4 ^b^	82.7 ^a^	84.1 ^a^	1.14	0.001	0.002	0.0008
**Ash**	42.7	40.1	45.4	47.2	1.16	0.067	0.050	0.161
**Ca**	38.0	35.1	42.0	40.1	1.04	0.268	0.363	0.404
**P**	52.8	54.1	55.6	55.4	1.02	0.860	0.378	0.645

A significant difference was considered at *p* < 0.05 based on Tukey’s HSD test. Trends toward significance (0.05 < *p* < 0.10) were also noted where applicable. Means within rows with different lower case letters differ significantly (*p* < 0.05). **NC**: basal diet without additives; **PC**: positive control (basal diet supplemented with 50 mg/kg bacitracin methylene disalicylate); **TOA-EOs**: basal diet supplemented with a microencapsulated mixture of Thyme, Oregano, and Ajwain. **DM:** dry matter, **CP:** crude protein, **Ca:** calcium, **P:** phosphorus.

## Data Availability

The original contributions presented in this study are included in the article. Further inquiries can be directed to the corresponding author.
